# A Pocket Medical Dictionary

**Published:** 1893-02

**Authors:** 


A Pocket Medical Dictionary, giving the Pronunciation and Definition of about 12,000 of the Principal Words used in Medicine and the Collateral Sciences. By George M. Gould, A. M., M. D., Author of a New Medical Dictionary; Ophthalmic Surgeon to the Philadelphia Hospital. Including a very complete table of the Arteries, Muscles, Nerves, Bacteria, Bacilli, Micrococci, Spirilli, and Thermometric Scales, and dose list of drugs and their preparations, in both the English and metric systems of weights and measures. Philadelphia: P. Blakiston, Son & Co. 1892.

In the preface to this useful book, Dr. Gould says that medical students and practitioners often have need of a small elementary word-book that may be slipped into the pocket for hurried reference and to serve as a passing reminder of the more commonly used terms. The compiler of this admirable condensed medical dictionary has expressed a great truth in the foregoing excerpt from his preface, and his book admirably fills the place which it is designed to occupy. In short, we consider this the best condensed medical dictionary that we have ever met with, and hesitate not to recommend it for the purposes it aims to meet. We particularly commend the editor for having eliminated diphthongs wherever practicable.

